# Supplementary material for: Are carbon emissions trading and green financial instruments synergistic? -Comprehensive quantitative research based on content analysis
Source: PLoS One. 2024 Mar 7;19(3):e0298601. doi: 10.1371/journal.pone.0298601 (PMC10919601; doi:10.1371/journal.pone.0298601)
Supplement: S1 Data — (ZIP) [file pone.0298601.s001.zip › Data/地级市Z_F工作报告.zip!/╣π╢1⁄2/╔╪╣╪╩╨/└┤╫╘╣1⁄2╓┌║┼í╛┬φ┐╦╩2╛▌═°í┐_2017.docx]

各位代表：

我代表韶关市第十三届人民政府向大会作工作报告，请予审议，并请政协各位委员和其他列席人员提出意见。

本届政府工作回顾

2012年至2016年，市政府深入贯彻党的十八大、十八届三中、四中、五中、六中全会精神和习近平总书记系列重要讲话精神，在省委、省政府和市委的正确领导下，在市人大和市政协的监督支持下，牢记韶关发展使命，团结带领全市人民，改革创新，真抓实干，较好地完成了“十二五”规划和十三届人大历次会议确定的主要目标任务，实现了“十三五”规划良好开局，各项建设取得新成绩。

——经济实力显著增强。全市地区生产总值迈上千亿元台阶，2016年达1227亿元（预计数，下同），五年增长52.2%；人均生产总值4.17万元，居全省山区市首位；地方一般公共预算收入85亿元，五年增长57.7%；固定资产投资736亿元，五年增长56%；社会消费品零售总额635亿元，五年增长65.4%。

——经济结构持续优化。规模以上工业增加值中，钢铁和有色金属两大传统产业占比由33%调整到14.1%，中央和省属企业占比由55%调整到39.3%，产业园区占比由15.5%提高到39.8%。县域经济和民营经济分别占全市生产总值的54.1%和52%，成为推动经济发展的重要力量。

——交通区位日益凸显。五年新增高速公路202公里、普通铁路117公里，改造国省道128公里，全面实现县县通高速公路，在粤东西北地区率先建成环城高速公路，形成了连接珠三角城市群的高快速通道，整体纳入大珠三角两小时生活圈。

——城市新格局初步成形。市区建成面积扩展到99平方公里，常住人口103万人。芙蓉新区建设全面提速，老城区、芙蓉新城和曲江城区一体化发展加快，长期以小岛为中心的城市格局正在改变。成为第三批国家新型城镇化综合试点城市。

——生态环境更加优美。全市森林覆盖率74.95%、有林地面积2128万亩、活立木蓄积量8822万立方米，均居全省首位。空气质量优良率达93.4%。饮用水源水质达标率100%。跻身国家生态文明先行示范区行列，通过国家园林城市复查，成功创建省林业生态市。

——民生福祉不断改善。民生支出797.4亿元，五年增长3.53倍。城镇、农村居民人均可支配收入达2.56万元和1.27万元，五年分别增长63.4%和71.2%。实现社会保险制度全覆盖，基本医疗保险参保率达98%以上。在全省山区市率先普及15年基础教育。22万贫困人口实现脱贫，贫困发生率从10.87%下降到5.16%。

五年来，市政府围绕建设粤北区域中心城市，按照“绿色转型、振兴发展”的思路，实施“五大战略”，开展“百项工程兴韶关”系列活动，强力推进“三大抓手”和对口帮扶。积极适应经济发展新常态，大力推进供给侧结构性改革。面向“十三五”，按照“一一二三八”的经济发展思路，积极实施主动融入珠三角总战略，开展“三大主题”工作，促进经济社会持续健康发展。

一、狠抓“三大抓手”，不断夯实发展基础

坚持以“三大抓手”为核心工作，全力以赴打响振兴发展攻坚战。加强交通基础设施建设，乐广、大广高速公路和赣韶铁路建成通车，武深、汕昆高速公路和北江航道扩能升级工程动工建设，韶新、雄乐高速公路和韶柳铁路开展前期工作，韶关丹霞机场项目取得突破性进展。推进产业园区扩能增效，省级产业转移园实现县域全覆盖，规上工业增加值达132亿元，是2011年的3.5倍，成为全市重要增长极。深化莞韶对口帮扶，由东莞市主导或协助引进产业项目达322个，完成投资820亿元。黄沙坪创新园进驻企业68家，成功入围省首批“互联网＋”小镇。推进中心城区扩容提质，芙蓉新城“三纵三横”主干路网基本成型，5个安置新村建设全面推进，商住小区初显规模，广东省韶关实验学校建成开学，商气人气明显集聚。大力开展“三旧”改造，实施项目32个。全力推进棚户区改造，建成棚改房2.79万套。在主城区东部初步形成可容纳10万人的城市新组团。实施一批“三江六岸”美化、绿化、亮化工程，城市功能稳步提升，面貌焕然一新。

二、加快转型升级步伐，着力优化产业结构

坚持优化存量与引进增量并举，实施先进装备制造、大农业、大旅游、大物流等八大产业发展三年行动计划，大力培育特色产业。五年累计签约项目2350个、开工项目5544个、投产项目4489个、新增“四上企业”857家，完成工业技改投资324.3亿元。先进制造业增加值、高技术制造业增加值分别达102.6亿元、15.6亿元，五年分别增长29%和1.3倍；民营工业增加值140.8亿元，五年增长1.8倍。新增国家4A级景区5家，2016年旅游收入和旅游者人数达324.7亿元和3582.6万人次，五年分别增长108.3%和64.5%。新落户金融机构18家，翁源农信联社成功改制农村商业银行，2016年全市存贷款余额分别达1630亿元和765亿元，五年分别增长56%和82.7%，存贷比由42.3%提高到46.9%。第三产业增加值624亿元、五年增长60.3%。粮食生产稳步增长，新增1个国家现代农业示范区、21家省级以上农业龙头企业和37件省名牌农产品，农业增加值达到167亿元、五年增长24.2%。三次产业结构由2012年的13.8:41.3:44.9调整为13.6:35.5:50.9。

三、深入推进改革创新，有效激发发展活力

坚持把改革创新作为推动振兴发展的不竭动力，坚决破除体制机制障碍，释放改革红利。深化“放管服”改革，制定行政许可事项目录，市级行政许可事项压缩至327项。市直49个部门全部制定并实施权责清单。开展“一门式一网式”政府服务模式改革，政府投资项目审批时限由273天压减到92天以内。改革商事登记制度，实行“多证合一、一照一码”，新登记市场主体90343户。深化国资国企改革，实现39户国有“僵尸企业”出清，国企利润增长36%。改革投融资体制，设立9只投资基金、总规模达139亿元。深化农村综合改革，完成集体林权改革，开展农村土地承包经营权确权登记颁证。出台创新驱动发展“1+N”政策，80家企业通过高新技术企业认定，专利申请量和授权量连续十年居全省山区市前列，荣获国家科技进步考核先进市称号。

四、坚持绿色发展引领，更加突显生态优势

坚持绿色发展理念，坚守生态红线，努力把生态优势转变为发展优势。实施主体功能区规划和环境保护规划，所有县（市）已纳入国家重点生态功能区。落实新一轮绿化广东大行动，实施《关于切实加强全市封山育林工作的议案》，五年造林204.5万亩，建成生态景观林带473.5公里。建成国家和省级自然保护区13个、森林公园81个、湿地公园10个。推进碳普惠制试点，成为全省唯一的碳排放权交易试点城市。综合治理石漠化，新增森林面积60多万亩。实行最严格水资源管理制度，连续三年获省考核优秀等次。加强对重点工矿企业重金属污染综合整治和环境修复，完成4个防控区重点工程，大宝山污水处理扩建工程投入运行。五年淘汰落后水泥产能488万吨、钢铁产能278万吨、蓄电池产能17.5万千伏安时，淘汰“黄标车”及老旧汽车1.94万辆，纳入国家节能减排财政政策综合示范城市。市区生活垃圾无害化处理率、城镇生活污水集中处理率分别达100%和80%以上。开展乡村清洁美工程。建成610个乡村绿化美化示范点。

五、大力实施民生工程，全民共享发展成果

坚持调整优化支出结构，集中有限财力保障和改善民生。每年承诺为民办实事，五年办了45件民生实事，努力解决住房难、看病难、出行难等群众普遍关心的突出问题。狠抓扶贫开发“双到”工作，五年落实扶贫资金40亿元，改造低收入困难户住房和农村危房3万户，搬迁“两不具备”贫困村庄277条、7551户，贫困村生产生活条件明显改善。大力开展市区基础教育资源布局调整，理顺办学体制，成功创建省教育强市，实现省教育强县和国家义务教育发展基本均衡县（市、区）全覆盖，高考综合成绩位居全省欠发达地区前列。优化配置市区医疗卫生资源，县级公立医院全部取消药品加成，完成基层医疗卫生机构综合改革，开展分级诊疗试点工作，推进人口计生优质服务。扩大就业和促进再就业，新增城镇就业24.5万人，完成劳动力技能晋升培训9.2万人，转移就业22.7万人。建立完善城乡居民大病保险制度。城镇、农村居民低保补贴分别提高到每人每月485元、335元以上，五年分别增长1.77倍和1.93倍。新开工保障房4.69万套，基本建成3.2万套。实施文化惠民，设立文化建设专项资金，建成一批文化场馆，县（市、区）数字影院、行政村农家书屋实现全覆盖。开展“群众点单、政府买单”的文化活动。完成142个中心村电网升级改造、2750公里农村公路硬底化、1186公里山区中小河流治理。建成95个乡镇农民体育健身工程，免费向社会开放96.2万平方米公共体育场地面积。深入推进“平安韶关”建设，全面加强社会管理综合治理。落实安全生产和食品药品监管责任，安全形势稳中趋好。前移审计关口，建立科学高效监督机制。依法依规做好统计工作，统计数据质量不断提高。成功创建第九届全国双拥模范城，连续两届获得“全国文明城市”提名资格。民族宗教、外事侨务、档案史志、新闻出版、气象水文、防震减灾、人防应急、妇女儿童、工会、残联、对台等工作取得新进步。

六、切实转变政府职能，加快建设法治政府

坚持依法行政、从严治政，努力建设人民满意政府。严格执行中央“八项规定”和国务院“约法三章”，扎实开展党的群众路线教育实践活动、“三严三实”专题教育和“两学一做”学习教育。坚持向市人大及其常委会报告工作、向市政协通报工作情况，注重发挥民主党派、工商联、无党派人士和人民团体的作用。五年办理人大议案1件、代表建议363件、政协建议案26件、政协提案635件，办复率100%。落实重大行政决策法定程序、咨询论证和政府法律顾问制度，我市成为全省首批被授予地方立法权的设区市。完成“六五”普法，建成四级公共法律服务中心（站），实现法律顾问村（社区）全覆盖。完成市县两级政府机构改革，精简议事协调机构374个、事业单位154个，精简率分别为80.4%和45.4%。开展办公用房专项清理工作。完成公务用车改革。开展市县镇村四级政务服务平台建设，乡镇便民服务中心实现全覆盖。强化廉政建设，成立市预防腐败局。

2016年是“十三五”开局之年，市政府做了大量谋划性、基础性、开篇性和突破性的工作，着力稳增长、促改革、调结构、惠民生、防风险。在全国全省经济增速放缓和我市经济下行压力不断加大的情况下，全市地区生产总值增长6%、在全省排位稳中有进，固定资产投资增长5%，地方一般公共预算收入增长2.28%（按可比口径），社会消费品零售总额增长9.3%。一是主动转变发展思路。在大珠三角经济区的格局下谋划韶关发展，积极与珠三角城市开展理念、交通、产业、市场、人才等方面对接，主动融入珠三角，成功纳入珠江西岸先进装备制造产业带。二是全面推进体制机制改革。启动了近年来涉及面最广、系统性最强、力度最大的体制机制改革，在行政审批、市与区财事权、投融资、国地税征管、教育、医疗卫生等方面取得重大突破。实施供给侧结构性改革，化解钢铁产能203万吨，超额完成商品房去库存任务，为企业减负28.8亿元，补短板基础项目完成投资120亿元。三是打造重大产业共建平台。与东莞共建华南先进装备产业园，为我市对接珠江西岸产业提供发展载体。积极谋划珠江东岸产业合作区，为推进大珠三角产业共建提供载体。谋划了“华南数谷”、空港产业园等一批打基础、利长远的重大平台，为我市产业发展厚植基础条件。四是开展招商引资培育经济新增长点。围绕装备制造、钢铁等传统优势产业，开展精准招商，引进和培育大数据、新能源汽车、先进装备等新兴产业，补齐补强产业链，推进产业转型升级提质。全年签订项目300个，总投资250亿元。五是破解融资瓶颈。充分用好用足用活国家政策性银行贷款，推动PPP项目，破解融资难融资贵问题，成功实施政府购买服务融资项目18个总额达220亿元，韶新高速和曲江大道成功通过PPP模式向社会融资，政府与社会资本合作实现零的突破。六是强力推动城市整体提升。围绕提升中心城市竞争力和市民认同感自豪感，按照既要“补短板”、又要“抢高点”的思路，启动城市整体提升三年行动计划，实施“五大提升工程”，计划三年投资300亿元、实施133个项目，力争“年年见成效，三年换新貌”。全年完成投资52.6亿元，开工项目47个、竣工24个，促进城市更加宜居宜业。七是突出抓好精准扶贫、教育现代化和卫生创强“三大重点”民生，统筹推进市区教育卫生资源整合，提升全市基本公共服务均等化水平。

本届市政府取得的工作成绩，离不开历届市委市政府打下的坚实基础，是全市上下共同努力的结果。在此，我代表市人民政府，向全市广大干部群众和企业家、驻韶部队官兵、各民主党派、人民团体以及社会各界人士，致以崇高的敬意！向所有关心和支持韶关振兴发展的港澳同胞、台湾同胞、海外侨胞和国际友人，表示衷心的感谢！

同时，我们清醒地看到，我市经济总量小，产业支撑力不强，民营经济薄弱，加强产业共建、加快经济发展任重道远；中心城区辐射带动能力不强，县域基础薄弱，城乡差距仍然较大，完善城市功能布局、促进城乡一体化任重道远；基本公共服务不够完善，民生和社会建设领域欠账较多，改善民生、发展社会事业任重道远；制约发展的陈旧观念和体制机制仍有不少，创新环境不优，人才聚集难，干部作风有待进一步转变，深化改革、优化发展环境任重道远。对此，我们将高度重视并认真解决，努力把各项工作做得更好。

新一届政府奋斗目标和主要任务

市第十四届人民政府履职期间，正是全面建成小康社会的决胜阶段，是我市主动融入珠三角、实现振兴发展的关键时期。纵观国内外形势，世界经济在艰难复苏，新一轮科技革命和产业变革蓄势待发，国内经济区域发展面临深度调整。我省区域开放合作日趋紧密，珠三角地区发展动力正在切换，对环珠三角城市的扩散效应逐渐增大，全面对口帮扶工作更加深入，“9＋6”大珠三角经济区，以及珠三角与粤东西北一体化发展新格局加快形成，我市发展所面临的格局正发生深刻变化。我们相信，只要在新一轮发展中主动融入珠三角，充分发挥我市交通区位优越、工业基础较好、发展腹地广阔的后发优势，坚持改革创新、真抓实干，今后五年将是韶关振兴发展的重要战略机遇期。

市第十二次党代会敏锐又准确地把握了区域发展的新态势，全面吹响了践行新发展理念、主动融入珠三角，全面建成小康社会实现振兴发展的号角，为今后五年的政府工作指明了方向。今后五年政府工作的总体要求是：深入贯彻习近平总书记系列重要讲话精神，紧紧围绕“五位一体”总体布局和“四个全面”战略布局，牢固树立和落实“新发展理念”，坚持稳中求进工作总基调，适应把握经济发展新常态，坚持以提高发展质量和效益为中心，以供给侧结构性改革为主线，坚定不移主动融入珠三角，强力推进“三大主题”工作、“三大重点”民生，加快韶关振兴发展，确保与全国同步全面建成小康社会，开启社会主义现代化建设新征程。

今后五年政府工作的主要目标是：以全面建成小康社会为核心任务，加快振兴发展步伐，力争地区生产总值年均增长10%左右，2020年全市小康指标指数达97%，全面建成小康社会；2021年地区生产总值突破2000亿元，人均生产总值达6万元以上，加快实现与珠三角一体化发展，建成更高质量的小康社会。

——交通条件提档升级。新增高速公路307公里，实现市到县通高速公路，国省道提升改造，韶关丹霞机场复航，北江航道扩能升级，建成跨赣粤桂的区域铁路通道，基本形成“八高四铁二航”的现代综合交通运输体系，融入珠三角一体化大交通格局。

——产业共建更加深入。传统优势产业巩固提升，新兴产业成为重要增长点，老工业基地实现转型发展，深度融入珠三角产业体系，建成广东重要的装备制造基地，与珠三角产业一体化发展。形成1至２个产值超500亿元的产业集群，基本建成现代产业新体系。

——城市功能大幅提升。芙蓉新区起步区初具规模，市区东部新城加快建设，曲江城区与主城区融合发展，市区建成区120平方公里以上，人口110万人以上。中心城区人居环境明显改善，城市竞争力整体提升。各县（市）新区基本建成，城镇体系进一步完善，全市户籍人口城镇化率达到50%。

——县域经济快速崛起。县域生产总值突破1200亿元，年均增长12%左右，经济总量占全市60%以上，特色产业体系基本形成。基本建成覆盖城乡的现代化基础设施体系，适度超前经济社会发展水平，县域发展迈入粤东西北地区前列。

——生态环境不断优化。节能减排任务全面完成，空气质量稳步提高，森林覆盖率、有林地面积、活立木蓄积量保持全省首位，成功创建全国生态文明先行示范区，打造广东绿色生态第一市，绿色生态优势进一步巩固提升。基本形成具有韶关特色的生态文明制度体系，资源节约型和环境友好型社会建设取得重大进展。

——民生福祉持续增强。城乡居民人均可支配收入达3万元以上，年均增长10%左右；现行标准下的贫困人口如期全部脱贫，贫困村贫困群众自我发展能力显著增强；建成城乡统一的社会保障体系；成功创建全国文明城市和省推进教育现代化先进市、省卫生强市；巩固发展双拥共建成果；公民科学素质明显提升，群众安全感明显增强。

围绕新一届市政府奋斗目标，着力完成以下主要任务：

一、坚定不移主动融入珠三角，构建一体化发展新格局

坚持把改革贯穿工作始终，在实现与珠三角一体化的格局下谋划韶关发展，学习借鉴珠三角先进经验，加强以市场为导向的改革，破除制约发展的思想和体制机制障碍。主动对接珠三角城市群发展规划，继续推进交通、园区等基础设施建设，加快实现与珠三角基础设施互联互通，谋划建设“华南数谷”、华南先进装备产业园、珠江东岸产业合作区和五大物流基地等重大产业平台，高标准打造参与珠三角乃至华南地区产业共建的重大平台，成功创建国家级高新区。打造大珠三角先进制造业基地、珠三角现代服务业的后援中心和高端农产品生产基地，建立公共资源互联共享机制，推动交通、产业、政策与珠三角一体化，推动基本公共服务均等化，在加快与珠三角一体化进程中实现振兴发展。

二、坚定不移推进供给侧结构性改革，全力振兴实体经济

坚持以满足需求为最终目的、以提高供给质量为主攻方向、以深化改革为根本途径，着力优化存量、引导增量、有效减量，全面完成省下达的“三去一降一补”任务。注重运用市场机制、经济手段、法治办法，完善企业退出机制，积极稳妥化解过剩产能，提高国有资本配置效率。多措并举打通供需通道，有效化解房地产库存，促进房地产市场健康发展。加强政府债务管理，防范政府债务风险，维护地方金融稳定。综合施策降低企业成本，构建普惠性政策扶持体系，优化实体经济发展环境。深入实施创新驱动发展战略，强化高新技术企业培育、新型研发机构建设、企业技术改造、孵化育成体系建设、创新人才队伍等工作，R&D经费占GDP比重提高到2.4%以上。大力推进大众创业万众创新，加强与珠三角协同创新，提升钢铁、烟草、有色金属、能源电力、建材等传统产业竞争力，战略性新兴产业占规上工业比重15%以上，民营经济占比提升至60%以上。精准发力，扩大有效投资，补齐产业项目、城市建设、县域经济、基础设施、社会民生等方面的短板，增加有效供给。

三、坚定不移抓好三大主题工作，增强发展新动能

深入推进项目攻坚，推进与大珠三角产业共建，构建与珠三角同一体系、不同环节的产业分工协作体系。发挥钢铁等基础材料、装备制造和农业生态等方面优势，巩固提升传统优势产业，培育壮大新兴产业，大力发展特钢产品，形成300万吨的特钢精品基地。五年新签约项目总投资1800亿元以上、新签约项目动工率60%以上、新签约超亿元产业项目600个以上。以“三规合一”为基础推进多规融合协调，构筑“一带两翼三心”城市空间发展格局，提升规划建设管理水平，打造大珠三角城市群北部战略节点城市。坚持“显山露水、用好山水”的思路，推进以“青山、碧水、田园”为重点的生态系统建设，彰显山水城市风貌。加快芙蓉新区和市区东部新城建设，推进曲江城区与主城区融合发展。完善基础设施和公共服务功能，整治“城市病”，改善人居环境，建设智慧城市，提升中心城市综合竞争力。围绕“一县一特色、一园一集群”，推进国家新型城镇化综合试点工作，加强县城、中心镇和全国重点镇建设，加强分类指导和差异化发展，激发县域发展活力。五年培育打造30个市级特色小镇，力争成功创建黄沙坪“互联网+”小镇等3个以上省级特色小镇。

四、坚定不移恪守生态底线，推进绿色低碳发展

深入实施主体功能区规划，加强生态建设和环境保护。大力发展现代农业、生态旅游、生命健康等绿色生态产业，将生态优势转化为发展优势，将生态产业培育成为新的经济增长点，实现“从绿掘金”。加强节能减排降耗工作，完成省下达的年度任务。加强大气、水、土壤污染防治。空气质量优良率不断提高。完成流经县（市、区）城区主要河涌整治，优良水质断面比例达85%以上、水功能区达标率达95%。全面治理农业面源污染。加强城市污水和农村垃圾处理，城镇生活污水处理率达85%以上，农村生活垃圾无害化处理率达75%。深入推进全国生态文明先行示范区建设，扎实开展新一轮绿化广东大行动，建设广东绿色生态第一市，森林覆盖率达75%以上，加快美丽韶关建设步伐。

五、坚定不移保障和改善民生，增强群众的获得感

拓宽共享发展道路，围绕民生事业发展短板加大投入，民生支出占比保持在75%以上，提高基本公共服务均等化水平。努力实现居民人均可支配收入年均实际增长和经济增长同步。坚决打赢精准扶贫、精准脱贫攻坚战，确保现行标准下的农村贫困人口在2018年全部脱贫。实施更加积极的就业政策，努力促进扩大就业。建立更加公平更可持续的社会保障制度，基本社会保险覆盖率稳定在98%以上。提高底线民生保障水平，大力发展慈善事业。坚持教育优先发展战略，深化教育综合改革，统筹教育资源配置，推进教育布局调整，促进各类教育协调发展，2018年成功创建省推进教育现代化先进市。深入实施12项国家基本公共卫生服务项目。推进城市公立医院综合改革，加快市级医疗资源整合和布局调整，全面提升基层医疗卫生服务能力，推进市县、县镇和镇村三个一体化，到2020年实现人人享有基本医疗卫生服务，2021年成功创建省卫生强市。构建现代公共文化服务体系，打响韶关特色文化品牌。广泛开展全民健身活动，城乡普遍实现15分钟健身圈。加强法治政府和精神文明建设，人民群众安全感和幸福指数明显提升。全面补齐单位生产总值能耗、每万人拥有律师数、文化产业增加值占生产总值比重等13项小康社会短板指标，确保2020年与全国同步全面建成小康社会。

2017年工作意见

2017年是新一届市政府的履新之年，也是保障“十三五”规划目标顺利完成的关键之年。今年经济社会发展的主要预期目标是：地区生产总值增长8％；固定资产投资增长12%以上；社会消费品零售总额增长10％；地方一般公共预算收入与经济增长基本同步；城乡居民人均可支配收入增长8%；居民消费价格指数涨幅控制在3%左右；城镇登记失业率控制在3.5%以内；R&D经费占GDP比重达1.2%；完成省下达的节能减排任务。

为实现上述目标，我们将重点做好以下工作：

一、强化体制改革，破除发展瓶颈

坚持以供给侧结构性改革为主线，以基础性、关键性改革为牵引，主动对接珠三角的思想观念和体制机制，进一步深化改革，构建高效的政务环境和良好的营商环境。

深化供给侧结构性改革。深入推进“三去一降一补”，实现更高水平的供需平衡。采取分类处置方式，实现全市国有“僵尸企业”基本出清、非国有“僵尸企业”处置有序推进。以实施去产能行动为契机，大力引入先进产能，全年承接珠三角地区转移项目50个，省级产业园完成工业增加值达到160亿元。加大不良贷款清收力度，鼓励银行业金融机构加大对中小微企业和“三农”的支持力度，确保金融机构杠杆率控制在合理水平。加大棚改货币化安置力度，积极培育商品房屋租赁市场，促进农村转移人口在城镇落户，力争全年化解商品房库存20万平方米。全面落实减税降费政策，打好降低企业制度性交易、人工、社会保险、财务、物流等成本组合拳，进一步减轻企业负担。

深化行政体制改革。加大“放管服”改革力度。推进“一门式一网式”政府服务模式改革，推动网上办事大厅的建设应用，构建市县镇村四级政务服务体系。理顺市与市辖区权责关系，建立事权和支出责任相适应的财政制度，推动管理和服务重心下移。健全“多规合一”基础数据和业务审批平台。完善园区管理体制，促进产城融合。落实丹霞山管理体制改革。建立重点项目和招商引资项目用地预审制度，强化土地供应后续监管。健全公共资源交易管理体制。推进社会保障体制改革，多渠道解决养老保险资金缺口问题。加强社会信用体系建设，力争在政府采购等六项重点领域实现突破。推进文化体制和生态文明体制改革。

深化经济体制改革。深化商事登记制度改革，健全企业经营异常名录管理及“双随机”抽查监管机制，强化事中事后监管。建立现代产权制度，清理和废止各种影响投资者产权保护的制度。清理整顿“红顶中介”，切断中介机构与行政机关的利益关联。深化国地税征管体制改革和税制改革。推进农信社改制工作。落实促进民营经济发展的政策，消除对非公有制经济的隐性壁垒和差别待遇。

深化国资国企改革。加快国资监管部门职能转变，推进国有资产监管全覆盖。健全有效制衡的公司法人治理结构，构建灵活有效的市场经营机制。整合盘活国有资产，转化为国有资本。引进战略投资者参与国企改革，推进国资国企按板块组建集团公司，支持有条件的国企改制上市或发行企业债券，积极开展资本运营，做大做强政府投融资平台。稳妥发展混合所有制经济，支持国企和集体、民营、外资企业相互投资参股、联合组建公司。

深化人才体制改革。深化人事制度改革，增强事业单位用人自主权。实施“双百”人才工程，落实引进培养紧缺适用人才政策，优化人才环境。深化收入分配制度改革，积极搭建人才引进平台，发挥人才基金的撬动作用，完善人才服务机制，健全人才经费投入和保障机制，推动产业发展和公共服务领域的人才集聚。

二、强化投资拉动，做大经济总量

抢抓政策机遇，发挥投资对经济增长的关键性作用，持之以恒抓项目、抓投入，扩大有效投资的关联效应、乘数效应、结构效应，引导社会投资尤其是民间投资，增强经济发展后劲。

努力拓宽投融资渠道。优化政府投资方向，通过投资补助、基金注资、担保补贴、贷款贴息等方式，引导社会资本扩大投资。健全PPP项目工作机制，全力推进8个PPP项目组织实施，抓紧推出一批新的PPP项目。继续抓好政府购买服务项目政策性融资落地工作。用好各类产业基金，加大股权、债券等直接融资力度。发挥城投、交投、金投、旅投和东南五大公司的融资作用，建立市中小微企业信用信息和融资对接平台，落实省粤财公司在我市开展政策性融资担保工作，进一步缓解融资难题。支持各县（市、区）培育、发展和壮大政府融资平台，提升投资拉动能力。

持续狠抓招商引资。坚持把招商引资作为振兴发展的核心工作来抓，用好“一带一路”、珠西产业带等政策，从被动承接珠三角低端产业转向主动参与珠三角产业共建。实行市政府领导分区域带队招商和部门“一把手”招商。重点围绕先进装备制造、现代物流、大数据、现代农业、生态旅游等产业，在产业契合度高的城市设立招商代理处，开展驻点招商、中介招商、精准招商、以商引商和产业链招商。进一步理顺政府职能部门与县（市、区）、园区招商联动机制，落实好县（市、区）和园区的招商主体责任。加大乳源县少数民族税收优惠政策宣传力度，争取新增注册销售公司20家。开展以珠三角地区为重点的招商推介活动5场，新签约项目总投资280亿元以上、动工率50%以上。积极跟进龙浩集团、白云电器、浪潮集团、通用文具等重点招商项目，争取更多项目早日落地。

突出重点项目建设。加强项目调度工作，加快项目落地开工和竣工投产。实施重点项目66个，完成投资326.3亿元，其中工业项目15个、投资58.8亿元。全力推进重大基础设施建设，开工建设韶关丹霞机场和韶新高速，加快北江航道和武深、汕昆高速等项目建设，完成171公里国省道改造和50公里旅游公路建设。建成并投入运营京广高铁乐昌东站。投资6亿元推进新一轮农村电网改造。强力推进工业项目建设，建成国粤发电项目，加快南雄华电热电冷联供和韶能新丰生物质发电等项目建设，抓好韶钢提升改造等115项技改项目，完成技改投资80亿元，努力扩大制造业投资比重。深入实施中心城市“五大提升工程”，力争开工建设50个项目，完成投资110亿元，建成一批重大城建项目。

三、强化产业共建，提升集聚能力

深入实施项目攻坚三年行动计划，强化与珠三角产业共建，积极融入珠三角产业分工协作体系。

打造产业平台，完善共建载体。构建工业发展平台。推进产业园区扩能增效，促进园区内企业配套，实现园区新增投产工业项目14个，规上工业增加值增长12%。加快韶关高新技术产业开发区创建国家级高新区；高标准建设华南先进装备产业园，完善园区规划，健全基础配套，力争新引进企业20家，完成投资20亿元；在南部地区谋划建设珠江东岸产业合作区，积极承接珠江东岸城市的外溢产业和孵化企业；规划建设水电产业园，培育水电装备产业链。构建商贸物流平台。重点建设航空产业园、粤北国际物流中心、韶关港、东环线商贸物流产业带和公路物流园等项目，打造大珠三角重要物流基地，促进贸易转型发展。构建大数据产业平台。编制实施大数据产业规划，大力推进“华南数谷”建设，加快异地容灾备份中心、金融机构数据备份后援服务中心建设，组建大数据产业联盟。重点发展大数据云服务和衍生产业，将我市打造成广东大数据产业先行区。

致力转型升级，提升产业层次。坚持做特一产、做强二产、做旺三产的思路，推进产业融合发展，继续推进资源枯竭城市转型发展，培育新业态新经济，着力振兴实体经济。壮大先进制造业。大力发展宝钢特钢产品，积极为珠三角先进制造业提供优质特钢原材料；依托华南先进装备产业园引进汽车零部件、机械装备、紧固件、模具等产业，配套珠西先进装备产业。积极创建电力装备等优势产业联盟，引导企业“抱团出海”，参与国际产业分工。编制工业新增长点清单，力争投产项目80个，新增规上工业企业30户，全市规上工业增加值增长8%。发展现代服务业。突出环丹霞山旅游产业园和“九龄故里·百里画廊”乡村旅游示范线建设，推进南雄、翁源全国休闲农业与乡村旅游示范县建设，着力创建全国、全省全域旅游示范市，实现旅游收入和旅游者人数分别增长15%和18%。培育影视传媒、广告创意等文化企业和产业基地。鼓励珠三角地区金融机构来我市设立分支机构。新增限上商贸企业20家，批发零售业增加值增长7%以上。促进住宿餐饮消费，实现住宿餐饮业增长12%。推进农业现代化。围绕珠三角等地消费市场，深入推进农业供给侧结构性改革，狠抓农产品标准化生产、品牌创建、质量安全监管，增加绿色优质农产品供给。加强特色农业生产基地建设，重点打造南岭特色优质水果产业带、南岭茶叶产业带、特色烟叶产业带、粤北优质蔬菜和优质稻产区。

坚持创新驱动，激活内生动力。落实创新驱动发展“1+N”政策，组建7亿元的科技创业基金，鼓励企业自主创新。重点在装备制造、生物医药、新材料等领域加强产学研合作，规划建设大学科技园。推进黄沙坪创新园、紧固件产业园、创智城、创亿谷等科技孵化器建设。推动3家省级新型研发机构与我市企业开展合作。推行高新技术企业倍增计划，新增高新技术企业10家，实现高技术制造业增加值增长10%以上。大力推进质量强市建设，促进发展质量提升。实施“互联网+”行动计划，推动制造业与互联网融合发展，促进智能制造工程深入实施。

优化企业服务，构建新型政商关系。出台扶持政策，推动更多企业上市。落实市领导挂点联系企业制度，积极帮助企业解决实际问题。优化政务服务，完善投融资、检测检验等产业公共服务平台。支持大众创业万众创新，新登记初创企业户数增长8%。实施“一企一策”，促进宝钢特钢、韶能、东阳光等重点企业平稳增长。鼓励金融机构支持项目业主在韶关辖内金融机构融资贷款，存贷比提高到48%；搭建中小微企业信用信息共享平台，提高中小微企业融资成功率，金融业增加值增长7%以上。建立完善高成长小微企业名录库，重点培育扶持50家小微企业。完善“政企通”微信平台和企业诉求处理机制。

四、强化扩容提质，提升城市功能

实施城市整体提升三年行动计划，优化城市空间发展格局，加强城市基础设施建设，提升城市功能和管理水平。

加快芙蓉新区建设，打造北江明珠。坚持“五个优先”、“五个融合”的原则，加大政府主导项目投资力度，建立项目高效推进工作机制。加快推进地下综合管廊建设，启动新城水系活化工程，打造“海绵城市”。建成高铁站综合客运枢纽和百旺路、滨江路、芙蓉大道、芙蓉隧道等骨干路网。优先建设邻避设施，动工建设滨江公园、市民文化活动中心、市妇幼保健服务中心、三甲医院等一批公共服务项目，完善公交站场、路灯、农贸市场等配套设施。加强高铁站前片区、总部经济区和文旅休闲区的规划建设。推进芙蓉新城与莞韶产业园融合发展。

提升老城区功能，推进曲江融入主城区。实施老城提质系统工程，优化城市空间结构，严格控制新建项目，突出优化城市功能、改善城市形象。探索公共交通新模式，综合施策缓解交通拥堵问题；完善供水、供气、截污管网，加强社区体育公园和街边公园建设；实施城区老工业基地搬迁改造；抓好棚户区和城镇危旧房改造，推进市区东部新城适度开发；优化教育、卫生、文化等优质公共资源配置，引导小岛片区等过度密集地区的机构与人口向外疏解。加快江湾大桥和曲江大道建设，促进曲江城区与主城区融合发展；主城区部分文化体育功能向曲江区延伸，迁建市博物馆；推进曲江新城建设，打造服务大型工业企业和“大南华”的城市功能区，加速实现同城一体化。

加强功能配建，推进市区基础设施建设。实施交通顺畅、城市功能和环境与景观提升工程。加强道路建设，启动建设芙蓉北环山路、东堤北延长线，开工建设新兴路“小内环”和生态路东接莲花大道“大内环”。完成17个交通节点整治，建设一批公共停车场，新建优化一批公交站场，完成16个港湾式公交站改造，启动有轨电车首期示范线工程建设，谋划韶关火车东站新站项目。完成西河体育中心、市体育馆改造升级。加快“三江六岸”亮化工程建设。建设3大城市公园，疏理2条行洪通道，打造3个亲水平台，实施6条道路景观提升，全面优化城市出入口及内部景观环境。整饰广富新街、升平路历史街区，彰显城市文化特色。

强化服务管理，提高城市宜居水平。加强城市服务和管理体系智能化建设，建设智能交通管理系统，打造“智慧韶关”统一平台。推动“镇改街”和“村改居”工作。加快户籍制度改革。完善城市治理机制，实行“网格化”服务管理。严格维护公共空间，加强城市建筑物立面管理，严厉打击“两违”行为。推进城市市政基础设施、市政公用事业、便民服务设施等的市场化运营。

五、强化特色打造，做大县域经济

实施加快县域发展三年行动计划，加强县域发展统筹，实施差异化政策，努力塑造特色发展、错位发展新格局。

大力打造特色园区。坚持把园区作为县域经济发展的主战场，科学谋划产业发展定位，支持各县（市、区）以特色园区为依托，培育1至2个特色支柱工业产业，促进区域产业优势互补。完善园区功能布局，加强基础设施建设，提升园区承载力。完善配套政策，激发园区发展活力，全力提升园区产业集聚力和竞争力。着力做好项目引进和落地，壮大园区产业规模，打造各具特色产业集聚板块，实现县域规上工业增加值增长15%。

做大做强特色农业。加快国家现代农业示范区、粤台农业合作试验区翁源核心区和特色农业生产基地建设，重点抓好130万亩优质稻、30万亩高产油茶、60万亩水果、150万亩蔬菜、3万亩特色水产等绿色优质农产品基地建设。积极培育新型农业经营主体和服务主体，新增市级示范家庭农场50家以上、市级农民合作社20家以上、省市级农业龙头企业15家以上，实现农业增加值增长4.5%。推广农村电子商务，培育一批农产品专业市场电子产销平台，支持发展农产品物流平台，构建农业物流体系，拓宽农民增收渠道。

积极培育特色小镇。坚持规划先行、多规融合、文化引领，科学编制特色小镇发展规划。加强全市统筹协调，强化政府引导和服务保障，支持县域依托自身的产业特色、生态特点和文化特征，围绕单个产业打造生态圈，培育旅游休闲、商贸物流、生态农业、先进制造、“互联网+”和生命健康等特色小镇。

激发农村发展活力。落实承包土地“三权分置”，开展农村集体产权制度改革试点。推进国有林场、供销合作社改革。建立健全县域综合征信体系。创新农村普惠金融服务，开展涉农资金整合试点，建设政策性涉农融资担保平台，支持在农村增设金融机构服务点和设立村镇银行。建设县镇村互联互通的“三资”管理平台。改革农村公共服务体系和农村社会治理体系，推动村民自治和公共服务重心下移。

六、强化生态底线，建设美丽韶关

坚持“绿水青山就是金山银山”，强化主体功能区分区管控，实施各有侧重的绩效考核评价办法，建设全国生态文明先行示范区。

巩固绿色生态屏障。实施绿化造林三年行动计划，继续抓好森林碳汇造林、生态景观林带、森林进城围城、乡村绿化美化等重点生态工程建设，确保全年造林36.6万亩、森林抚育73万亩。加强封山育林，强化生态红线划定，严厉打击乱砍滥伐等林业违法犯罪行为。推进森林航空消防基地建设。发展森林生态旅游，建设一批森林公园、湿地公园。大力发展林下经济和林业产业。依托我市现有的产权交易平台，建立健全碳排放权、集体林权等权属的流转和交易机制，开发“绿色黄金”。

强化节能减排工作。鼓励清洁生产和资源综合利用，淘汰落后产能。推进化工、有色金属、铅蓄电池等行业实施效能提升、循环利用技术改造。扩大重点用能单位监管范围，推进能耗监控大数据管理平台建设。严格环保准入，实行污染物排放总量前置审核制度。加强污染减排工程建设，彻底清理禁养区畜禽养殖场。

注重环境保护治理。加强大气污染防治，强化机动车污染防控，推广新能源汽车200辆，淘汰“黄标车”及老旧汽车3200辆以上。强化工业废水治理，实施江河湖库水生态修复和饮用水水源保护工程，加快市区6万吨污水处理项目及雨污分流排水管网建设，实现城镇生活污水处理率达83%以上。抓好全国土壤污染综合防治先行区建设，开展土壤分级管理，推进大宝山、凡口等周边土壤治理与修复。加强园区污染防治，推进园区污染治理设施和集中供热工程建设。推动城市垃圾强制分类，加快市区生活垃圾处理设施规划建设。严格环境监管执法，严厉打击恶意排污、违法排污以及非法盗采矿产资源等违法行为。

改善农村人居环境。抓好五彩瑶乡等新农村示范片项目建设。实施农村人居环境综合整治“五项攻坚工程”，推进农村环境连片综合整治，实现全市20户以上自然村村庄整治覆盖率达50%。加快推进新一轮农村生活垃圾和污水处理基础设施建设，促进农村生活污水就近净化处理。积极推动生态示范镇创建，建成12个省级以上生态文明乡镇；建成156个乡村绿化美化示范点。新增296个行政村通自来水，实现全市行政村自来水覆盖率、农村生活饮用水水质合格率均达88%以上。加强历史文化名村、历史建筑、传统村落、古树名木保护。

七、强化公共服务，增进民生保障

坚持发展为民，优先安排民生支出，民生占比提高到75%以上，进一步提高基本公共服务支出占比，解决好群众普遍关心的突出问题。

突出“三大重点”民生。加快扶贫大数据平台建设，实施“五个一批”重点工程和“三个到户”项目，推进精准扶贫精准脱贫，实现3.35万相对贫困人口稳定脱贫。推进教育现代化建设、基础教育改革和市区教育资源整合，提高教学质量和办学水平，完成20间中小学和幼儿园的改扩建，力争创建4个以上省推进教育现代化先进县（市、区）。推进省卫生强市创建，启动18家城市公立医院综合改革，推进“三医”联动改革，扩大分级诊疗试点，建设纵向医联体、医共体，推动医疗服务重心下移和诊疗资源下沉，加强基层医疗卫生服务能力建设，推进市区2家医院整合。

持续增进民生福祉。大力促进创业带动就业，加强原北江技校、韶关学院和鑫金汇等创业孵化基地建设。推进基本医疗保险城乡一体化，完善异地就医即时结算程序，实现社保业务进村（社区）。加快农村危房改造，基本建成保障房2247套。加强被征地农民社保工作，提高城乡低保、农村五保救助保障水平。发展适度普惠型社会福利和慈善事业，保障特殊困难群众基本生活。培育养老服务多元主体，探索医养融合模式，试点敬老院公办民营。加快建设芙蓉新城文化组团等标志性文化场馆。落实文化惠民工程，建成10个示范性基层综合文化服务中心。加大文化遗产保护力度。建设市妇女儿童活动中心。推广全民健身活动，发展体育事业，擦亮广东户外运动天堂和南粤古驿道旅游品牌。完成397.7公里中小河流治理，做好病险山塘水库除险加固。完成99个中心村电网改造。在南雄试点的基础上全域推广超高速无线局域网应用，加快打造信息高速公路先行市。

推进社会治理创新。着力打造立体化、数字化的社会治安防控体系，深入创建“平安韶关”。加强矛盾纠纷排查，着力化解信访积案。加强公民思想道德建设。统筹推进创文巩卫申名工作。加强法治宣传教育，启动“七五”普法，完善“一村（社区）一法律顾问”制度。加强应急指挥平台和突发事件预警信息发布平台建设，提高公共安全和防灾减灾能力。完善安全生产监管体系，实行食品药品安全风险分级管理，维护市场交易和质量安全。

今年着力办好10件民生实事：1.完成南水水库供水一期工程；2.完成市区教育资源整合一期工程；3.完成基层医疗卫生服务能力提升工程；4.完成市区10个农贸市场升级改造；5.完成市区2000个公共停车位建设；6.完成市区旧堤加固和环境提升工程；7.完成市区公园绿地10万平方米；8.完成5600户农村危房改造；9.完成农村公路硬底化500公里以上；10.实施农村生活垃圾治理工程。

八、强化政府建设，提升服务能力

落实法治政府建设实施纲要，加强政府建设，不断提升政府公信力执行力，着力打造法治型、高效型、廉洁型政府。

坚持依法行政，打造法治政府。自觉接受人大及其常委会和人民政协、新闻舆论、人民群众的监督，认真办理人大代表议案、建议和政协委员提案，主动听取社会各界的意见建议。加强生态环境保护、城市升级改造、岭南历史文化传承与保护等方面立法工作。实施重大行政决策目录管理。加强新型智库建设。坚持严格规范公正文明执法，强化对行政权力的制约。深化政务公开。加强行政复议和行政应诉，完善行政争议协调化解机制。

坚持勤勉从政，打造高效政府。加快转变政府职能，加大简政放权力度，优化政府组织架构，不断提升行政效能。建立科学高效的督查督办制度，加强现场办公和现场督办。加强公务员队伍建设，强化干部担当精神。继续改进文风会风。加强监督管理，加强12345政府服务热线建设，落实行政许可年度报告制度，逐步建立健全审批、管理、监督运行机制。

坚持从严治政，打造廉洁政府。落实中央八项规定精神和“两个责任”实体制度，将“三重一大”事项纳入风险和可控性评估管理，从源头上预防和惩治腐败。强化对财政资金分配使用、国有资产监管、政府投资、政府采购、工程建设、公共资源转让等重点领域的监督。加强机关作风建设和效能问责，开列“负面清单”，推动责任落实和作风转变。加强行政程序制度建设，推进廉政风险防控工作全覆盖。加大行政监察和审计监督力度，严肃查处各类违纪违法案件。切实纠正部门和行业的不正之风，着力解决损害群众利益的突出问题。

各位代表！功崇惟志，业广惟勤。主动融入珠三角，全面建成小康社会，实现振兴发展，是我们共同的责任和担当。新的航程已经开启，新的任务光荣艰巨。让我们在市委的坚强领导下，紧紧依靠全市广大干部群众，真抓实干、不忘初心、继续前行，为加快实现韶关振兴发展作出更大努力！
